# Pediatric Respiratory Support Technology and Practices: A Global Survey †

**DOI:** 10.3390/healthcare5030034

**Published:** 2017-07-21

**Authors:** Amélie O. von Saint André-von Arnim, Shelina M. Jamal, Grace C. John-Stewart, Ndidiamaka L. Musa, Joan Roberts, Larissa I. Stanberry, Christopher R. A. Howard

**Affiliations:** 1 Department of Pediatrics, University of Washington and Seattle Children’s, 4800 Sand Point Way NE, M/S FA.2.112 P.O. Box 5371, Seattle, WA 98105, USA; shelinajamal15@gmail.com (S.M.J.); Ndidi.Musa@seattlechildrens.org (N.L.M.); joan.roberts@seattlechildrens.org (J.R.); 2 Departments of Global Health, Medicine, Epidemiology, and Pediatrics, University of Washington, 325 9th Avenue, Box 359909, Seattle, WA 98104, USA; gjohn@u.washington.edu; 3 Seattle Children’s Research Institute, Seattle, WA 98101, USA; larissa.stanberry@gmail.com (L.I.S.); christopher.howard@seattlechildrens.org (C.R.A.H.)

**Keywords:** oxygen, respiratory technology, mechanical ventilation, non-invasive mechanical ventilation, international health

## Abstract

**Objective:** This global survey aimed to assess the current respiratory support capabilities for children with hypoxemia and respiratory failure in different economic settings. **Methods:** An online, anonymous survey of medical providers with experience in managing pediatric acute respiratory illness was distributed electronically to members of the World Federation of Pediatric Intensive and Critical Care Society, and other critical care websites for 3 months. **Results:** The survey was completed by 295 participants from 64 countries, including 28 High-Income (HIC) and 36 Low- and Middle-Income Countries (LMIC). Most respondents (≥84%) worked in urban tertiary care centers. For managing acute respiratory failure, endotracheal intubation with mechanical ventilation was the most commonly reported form of respiratory support (≥94% in LMIC and HIC). Continuous Positive Airway Pressure (CPAP) was the most commonly reported form of non-invasive positive pressure support (≥86% in LMIC and HIC). Bubble-CPAP was used by 36% HIC and 39% LMIC participants. ECMO for acute respiratory failure was reported by 45% of HIC participants, compared to 34% of LMIC. Oxygen, air, gas humidifiers, breathing circuits, patient interfaces, and oxygen saturation monitoring appear widely available. Reported ICU patient to health care provider ratios were higher in LMIC compared to HIC. The frequency of respiratory assessments was hourly in HIC, compared to every 2–4 h in LMIC. **Conclusions:** This survey indicates many apparent similarities in the presence of respiratory support systems in urban care centers globally, but system quality, quantity, and functionality were not established by this survey. LMIC ICUs appear to have higher patient to medical staff ratios, with decreased patient monitoring frequencies, suggesting patient safety should be a focus during the introduction of new respiratory support devices and practices.

## 1. Introduction

Hypoxemia is a common complication of critical illness in childhood that may increase mortality. It is observed in both respiratory and non-respiratory diseases [1,2,3,4,5,6,7,8]. Severe hypoxemia leads to poor oxygen delivery to tissues, anaerobic respiration, tissue hypoxia and, if left untreated, eventually death [2,9]. Oxygen therapy and respiratory support for hypoxemia are critical components of international pediatric resuscitation guidelines [10,11,12,13]. Management of critically ill children with hypoxemia often includes high-cost interventions, staff with specialized training, and new technology. However, in regions with the highest burden of pediatric respiratory morbidity and mortality, these resources may be unavailable [14].

Data on pediatric respiratory support practices and resources, specifically respiratory technology availability for acute respiratory diseases globally, are limited. Research to improve existing respiratory technology interventions is necessary to help decrease hypoxemia and respiratory failure-related mortality in low-resource-settings [15]. To ensure successful translation of technology research into medical practice, data on human factors and device environment are important [16,17,18]. In the case of pediatric respiratory equipment these include facility infrastructure and staffing, commonly available equipment and expertise levels. A few adult reports [19,20] and one pediatric report [21] have started to address critical care services and utilization in Low- and Middle-Income Countries (LMIC); however, little is known about international differences in pediatric respiratory support systems. This global survey assesses the current respiratory support capabilities for children with respiratory illness in different economic settings.

## 2. Materials and Methods

An online, anonymous survey was developed to target medical providers with experience in managing children with acute respiratory illness. The survey included 11 mandatory sections, with 22 questions, and an optional section focused on respiratory scores. Questions were numeric, binomial, categorical or descriptive, and developed by the research team and reviewed by national and international clinical and research colleagues. Definitions of common terms like Intensive Care Unit (ICU) were not provided, and interpretation was left to the discretion of the survey respondent. The survey is available in the electronic supplement (Supplementary Materials). The survey was reviewed by the Seattle Children’s IRB with an exempt determination.

The survey was built and managed using REDCap software and accessed through the University of Washington. Convenience sampling was performed between December, 2014 and April, 2015 by distributing the survey to World Federation of Pediatric Intensive and Critical Care Society (WFPICCS) members (physicians, nurses, mid-level providers, and other health care professionals) via email. In addition, the survey was made available on the Pediatric Critical Care Medicine (PCCM) website, 99NICU online blog, and within the researchers’ personal networks. The electronic survey was open for approximately 3 months. While convenience sampling is not a preferred method for sampling populations, it enabled access to a geographically broad participant group, cost effectively, and within an acceptable time frame.

The collected data were divided into two segments, LMIC and HIC, based on 2015 fiscal year income levels as determined by the World Bank [22]. Data from Low, Lower-Middle, and Upper-Middle economic segments were combined to form an aggregate LMIC segment. The High Income economic segment was analyzed as the HIC segment. Survey data were analyzed using graphical and descriptive statistics in Microsoft Excel 2010 (Microsoft Corp., Redmond, WA, USA), Tableau Desktop 8.2 (Tableau Software, Seattle, WA, USA), and R (R Core Team, Vienna, Austria). The results were presented using summary and graphical statistics. Since the respondents’ identities and healthcare institutions were not recorded, the independence of the survey responses cannot be established conclusively. For example, some respondents could have filled out the survey multiple times, or respondents from the same institution could have provided duplicate or conflicting answers. Furthermore, since the number of LMIC economies represented in the survey was lower than HIC, we abstained from using any statistical tests as means of inference and instead relied on descriptive statistics only.

Not all participants finished the survey. Hence, the number of participants completing each survey section was counted and used to calculate the percentage of responses to the questions in that section. Participants were determined to have completed a section if they responded to one or more mandatory questions in a section.

## 3. Results

The survey was initiated by 357 individuals and completed (to the last mandatory survey question) by 295 (83%) participants (Table 1).

Of the 64 countries represented in the survey, 28 were High-Income countries (HIC), 36 were from Low- and Middle-Income Countries (LMIC). Geographically, 31 respondents were from Africa, 42 from Asia, 11 from the Middle East, 45 from Central and South America, 31 from Australia and New Zealand, 80 from North America, and 114 from Europe and Scandinavia. Survey participants working primarily in HIC provided 67% of the total completed surveys responses. The largest group of survey responders was physicians, the majority of whom were pediatric critical care physicians (Table 1). Urban public tertiary healthcare facilities represented the most common work environment of the respondents (Table 1). The mean number of ICU patients a medical provider takes care of at a time was higher in LMIC than HIC (Table 1). Hospital-acquired infections were tracked by the majority of facilities in LMIC and HIC. Paper-only medical records remained similarly widespread in 41% of HIC and 40% of LMIC, while exclusively electronic methods were utilized in 39% of LMIC and 33% of HIC, with the remainder using a combination of paper and electronic record systems. Electricity was reported to be available for 24 h/day in 99% of all surveyed centers (Table 1). Mobile phone connectivity was available in ≥80% surveyed facilities in both LMIC and HIC, while mobile data connectivity was present in 57% LMIC and 56% HIC.

### 3.1. Respiratory Support Capabilities

**Figure 1:**
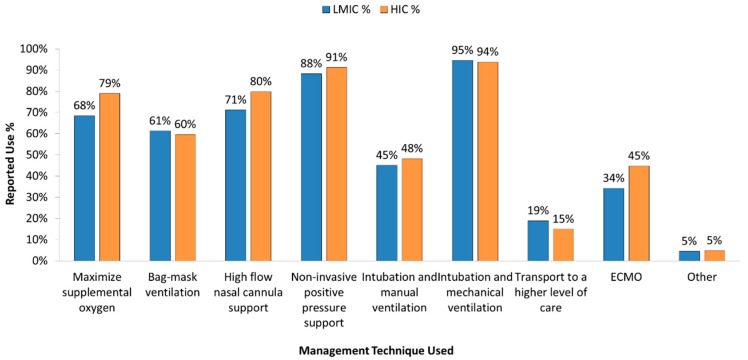
Management of Acute Respiratory Failure in different economic settings.

The most commonly identified form of respiratory support equipment used to manage acute respiratory failure was intubation and mechanical ventilation, followed closely by non-invasive positive pressure support (Figure 1).

**Figure 2:**
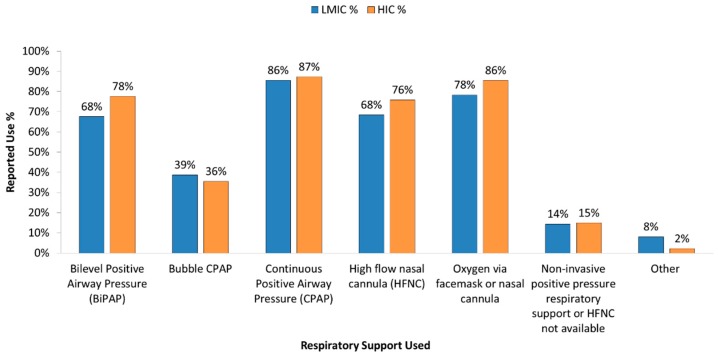
Forms of Non-invasive Respiratory Support used in different economic settings.

ECMO was used in 45% of HIC and 34% of LMIC for management of acute respiratory failure. Of the centers in LMICs reported to provide ECMO, 76% were located in Upper-Middle economies. Non-invasive positive pressure ventilation describes the delivery of mechanical respiratory support without the need for endotracheal intubation through an interface (nasal prongs or mask, face mask, or helmet) that delivers continuous positive airway pressure (CPAP) or bi-level positive airway support (BiPAP) [23]. CPAP was the most common form of non-invasive respiratory support used in this survey, followed by oxygen via facemask or nasal cannula (Figure 2).

While BiPAP availability was reported by 68% of respondents from LMIC, 69% of these were from upper middle-income countries. Bubble-CPAP is a simple, low-cost version of CPAP that generates positive-end-expiratory pressure by connecting the expiratory limb of a breathing circuit to a tube, which is submerged in water [24]. Bubble CPAP use was reportedly low in both LMIC and HIC.

High-flow nasal cannula oxygen therapy involves delivery of heated and humidified oxygen via special devices at higher flow rates than simple nasal cannula oxygen therapy. High-flow nasal cannula therapy (HFNC) was widely used (Table 2).

We did not inquire if HFNC was used with oxygen only or blended air. Other respiratory support devices reported in use or available included non-invasive neurally adjusted ventilatory assist (NIV NAVA), SiPAP, high frequency oscillation, and negative pressure ventilation.

### 3.2. Respiratory System Component Availability

Oxygen, air, gas humidifiers, breathing circuits, patient interfaces, positive pressure ventilation systems (e.g., CPAP, BiPAP, HFNC, ventilator), and oxygen saturation monitoring were reported to be almost universally available (Table 2).

The primary source of oxygen and medical air reported was wall outlet. Oxygen and medical air bottles, oxygen concentrators, and air compressors were less commonly reported to be available (Table 2).

In bottle or bubble humidifiers, gas is bubbled through a body of water that may or may not be actively heated, usually contained within a screw-top bottle [25]. Bubble or bottle humidification was used by over 50% of all respondents. Heat and moisture exchangers (HME) capture and return the heat and moisture produced during respiration back to the patient [26]. Their use was higher in HIC.

Bi-nasal prongs were the most commonly reported form of patient interface with 2–4 different types of interfaces typically available.

### 3.3. Factors that Promote the Ongoing Use of New Equipment and Technology for Survey Respondents

The factors most frequently perceived to be very important in promoting the ongoing use of new equipment included equipment safety, adequate training and support of doctors and nurses, and scientific (published) clinical evidence supportive of the intervention. Change in workload, ongoing costs (electricity, consumables, and maintenance costs), re-usability and durability of equipment components, and initial equipment cost were rated less highly, even in LMIC (Table 3).

Most responders indicated that their primary work facility had successfully implemented standardized clinical protocols for management of common acute illness. Methods most helpful for implementation of standardized clinical protocols included adequate training and introduction to the new practice, ongoing support for the practice change, and availability of all necessary equipment.

### 3.4. Assessment and Charting of Respiratory Distress or Failure

The top methods of assessing a patient with respiratory distress were oxygen saturation; work of breathing (defined as grunting, flaring, tracheal tugging); chest retractions; and blood gas analysis (Table 4).

The top four clinical parameters documented in patients’ charts were respiratory rate, oxygen saturation, heart rate, and fraction of inspired oxygen (FiO_2_). While the type of clinical assessments was similar in HIC and LMIC facilities, the frequency of documentation for these parameters was every hour in HIC versus every 2–4 h in LMIC facilities.

For survey respondents using respiratory scores for patient assessments (*n* = 101), the main elements used in these scores in descending order included respiratory rate, degree of chest retractions, oxygen saturation, work of breathing, auscultation findings, cyanosis, FiO_2_, heart rate, and mental status. Other reported monitoring used in LMICs included respiratory resistance and compliance, oxygen index, ventilator settings, end tidal CO_2_, and lab results. Other additional HIC monitoring included fluid balance, chest x-ray, nasal-gastric drainage, and the Paediatric Early Warning Score.

## 4. Discussion

This survey indicates some differences and many similarities in reported pediatric respiratory and critical care support system availability and use across 64 countries.

Among public, urban, tertiary care centers around the world management of children with acute respiratory failure appears intubation and mechanical ventilation was the most common form of support. Consistent with these findings, Tripathi et al. did not find significant differences in access to mechanical ventilators between different economic settings [21]. Our data showed expected differences in the use of extracorporeal life support of acute respiratory failure. ECMO was less available in surveyed centers from low and lower middle income countries, and is consistent with country registries of the Extracorporeal Life Support Organization (ELSO) [27].

While bubble-CPAP systems may be lower in cost and considered clinically preferable to machine CPAP, especially for neonatal patients [28,29,30,31,32], its use was infrequently reported in all economic settings. This may change with the increasing evidence supporting the use of bubble-CPAP in infants and children, especially in LMIC [33,34,35].

The reported widespread availability of wall oxygen, medical air, and oxygen saturation monitors in LMIC facilities was surprising; since medical oxygen reportedly is not widely or reliably available due to financial constraints, poor infrastructure, and inadequate capacity of supply management and equipment maintenance in these settings [36,37,38]. Particularly for pediatric patients, access to oxygen has been limited in LMIC facilities due to insufficient supply and competition for use by other services [39]. Using a simple model that links care pathways to the progression of pneumonia in young children, Floyd et al. predicted that a combination of pulse oximetry with current World Health Organization (WHO) Integrated Management of Childhood Illnesses (IMCI) guidelines has the potential to avert up to 148,000 deaths per year in the 15 countries with the highest burden of pneumonia across Africa and Asia, under the assumption that there is more than 90% prognostic tool and supplementary oxygen availability [40]. Hence, data on availability of oxygen and oxygen saturation monitors in these settings, even if limited to larger and better-resourced centers within LMIC matters.

The new WHO “paediatric emergency triage, assessment and treatment (ETAT) guidelines for emergency treatment of hypoxemia in limited resource settings” recommend the addition of effective heated humidification when flows of greater than 4 L/min through nasal cannulae are required for more than 1–2 h [41]. Development of more effective humidification systems may be necessary in LMIC where bubble humidifiers remain prevalent, despite evidence of their poor performance even in tropical climates [42]. Exploration of the role of humidified high-flow oxygen therapy in achieving better clinical outcomes for children presenting with respiratory distress or other emergency signs was determined to be a research priory by the WHO [41]. Reports of HFNC use in LMIC have been limited thus far [35]. The survey results suggest frequent use of HFNC in LMICs.

To have global impact, it is not sufficient to simply develop an affordable, effective respiratory support system. In addition, staff must be trained to deliver the therapy, clinical guidelines and monitoring for use must be in place [24]. According to survey respondents, ongoing use of new equipment is promoted by emphasis on clinical efficacy, scientific evidence, and safety of the devices, in addition to adequate training of clinicians, and provision of ongoing technical support, consistent with studies examining introduction of Bubble CPAP in low-resource settings [43,44]. Reusability, while understood to be prevalent, and anecdotally desirable, was not a key factor for promoting new technology use for survey respondents from LMIC. The initial equipment cost was also not rated highly by participants, likely due to the strong representation of better-resourced healthcare facilities in both LMIC and HIC.

Given the differences in health care financing and insurance schemes in LMIC economies, invasive respiratory support may be out of reach for the majority of patients in limited-resource settings [45]. However, cost-effective care, providing more-than-usual resources to rescue severely ill patients with acute respiratory failure and hypoxemia, has a fundamental place, and should be a fundamental goal in any health system [46].

Tripathi et al. reported almost twice the number of intensivists working in PICUs in resource-rich versus resource-poor regions (10 [SD 5.8] vs. 5.5 [SD 4.3], but did not assess the patient to provider ratios [21]). The results of this survey indicate that the patient to ICU physician and nursing staff is on average much higher than in HIC. Since patient monitoring is also less frequent in these settings, medical device safety and ease of use are especially important in LMIC.

This survey has a number of limitations. The key limitation is the population sample of mostly urban, tertiary care centers in both HIC and LMIC, which may not be representative of many facilities in Low Income countries. However, the fact that 72 centers from 12 countries in Africa and Asia respectively report comparable resources is encouraging. Additional responses from LMIC are needed. We did not provide definitions for the term “intensive care unit”, which could mean different care levels in different settings. It is possible that the survey design, its length, and language barriers were a deterrent to some participants. However, survey completion was estimated to take less than 15 min, and percentage of participant attrition was similar in HIC and LMIC. The survey results do not imply equipment functionality, availability in adequate quantities or adequate expertise available to run the equipment safely or effectively.

## 5. Conclusions

In conclusion, the survey results suggest many similarities in the availability of respiratory equipment, and care practices for children with hypoxemia and respiratory failure in public, urban, tertiary care centers worldwide. Given decreased health care provider staffing and patient monitoring in LMIC compared to HIC, focusing on safety of respiratory technology and implementation may impact especially children with respiratory diseases in LMIC. Even after accounting for the survey limitations, this effort advances our understanding of resource availabilities for critically-ill children globally, and may guide future research on respiratory technology for less-developed parts of the world.

## Figures and Tables

**Table 1:** Characteristics of survey respondents and their healthcare facilities in Low- and Middle-Income (LMIC) and High-Income Countries (HIC) (*n* = total number of responses; % = percent of total responses).

Characteristics of Respondents and Their Facilities		LMIC *n* (%)	HIC *n* (%)
**Number of participants *n* = 357**	Survey started	118	239
	Survey completed	100	205
	Survey attrition rate	18	34
**Geographic Representation *n* = 357**	Number of countries represented	36	28
**Occupation *n* = 357**	Nurse	7	59
	Respiratory Therapist	4	13
	Physician	105	163
	Other	2	4
**Facility setting *n* = 354**	Urban	101	209
	Suburban	8	21
	Rural	5	7
	Other	2	1
**Facility type *n* = 354**	Public	88	198
	Private	23	36
	Faith-based	3	1
	Other	2	3
**Facility level of care *n* = 353**	Primary	5	2
	Secondary	10	10
	Tertiary	96	223
	Other	2	5
**Bed types *n* = 352**	Neonatal ICU	86	170
	Pediatric ICU	74	196
	Mixed pediatric/adult ICU	17	30
	Adult ICU	32	56
**Mean number of patients managed by one health care provider at time *n* = 327**	*Physicians*		
	Emergency Room	22	19
	PICU	19	10
	NICU	26	13
	*Nurses*		
	Emergency Room	9	9
	NICU	5	2
	PICU	6	3
**Tracking hospital acquired infections *n* = 310**	Yes	94	192
	No	7	9
	Don't know	2	6

**Table 2:** Respiratory equipment use and availability in LMIC and HIC hospitals (*n* = total number of positive responses; % = percent of total responses). Survey participants could choose multiple responses.

Characteristics of Respiratory Support Available		LMIC *n* (%)	HIC *n* (%)
**Management of acute pediatric respiratory failure *n* = 339**	Maximize supplemental oxygen	76	180
	Bag-mask ventilation	68	136
	High flow nasal cannula support	79	182
	Noninvasive positive pressure support	98	208
	Intubation and manual ventilation	50	110
	Intubation and mechanical ventilation	105	214
	Transport to a higher level of care	21	34
	ECMO	38	102
**Non-invasive respiratory support used *n* = 339**	Bilevel Positive Airway Pressure (BiPAP)	75	177
	Bubble CPAP	43	81
	Continuous Positive Airway Pressure (CPAP)	95	199
	High flow nasal cannula (HFNC)	76	173
	Oxygen via facemask or nasal cannula	87	195
	Noninvasive positive pressure respiratory support or HFNC not available	16	34
	Other	9	5
**Availability and regular use of respiratory system components *n* = 322**	Oxygen source	106	216
	Air source	104	206
	Air and Oxygen	104	206
	Gas blender	92	178
	Gas humidifier or heater	101	196
	Breathing circuit or tubing	104	198
	Patient interface	106	201
	Positive pressure ventilation system (e.g., CPAP, BiPAP, HFNC, ventilator)	105	200
	Oxygen saturation monitor	106	202
**Oxygen source *n* = 322**	Wall outlet	104	211
	Bottles	51	120
	Oxygen concentrator	13	21
	Other	2	0
	Don't know	31	0
**Air source *n* = 322**	Wall outlet	101	198
	Bottles	29	74
	Oxygen concentrator	18	18
	Other	0	0
	Electric pump	1	4
	Don’t know	1	2
**Humidifiers used *n* = 322**	Bubble or bottle humidifier	56	122
	Pass over or wick humidifier	28	78
	Heat and moisture exchanger (HME)	55	143
	Other	2	2
	Don’t know	31	28

**Table 3:** Factors influencing ongoing equipment use (*n* = total number of “very important” responses; % = percent of total responses). Survey participants could choose multiple responses.

Factors rated “Very Important” in Ongoing Use of New Equipment (*n* = 310)	LMIC *n* (%)	HIC *n* (%)
Equipment safety	66	154
Adequate training and support of doctors and nurses	67	138
Scientific (published) clinical evidence	62	132
Personal experience	38	94
Durability of the equipment	38	69
User-friendliness of the equipment	32	65
Ongoing technical and maintenance support	30	61
Initial equipment cost	29	55
Workload of medical staff	25	54
Reusability of all equipment components	26	39
Ongoing costs (electricity, consumables, maintenance)	23	35
Use is required by a supervisor or manager	11	23

**Table 4:** Assessments of respiratory disease severity in LMIC and HIC. Survey participants could choose multiple responses.

Respiratory Assessment Method Used (*n* = 307)	LMIC *n* (%)	HIC *n* (%)
Oxygen saturation	98	202
Work of breathing (grunting, flaring, tracheal tugging)	97	202
Chest retractions (degree, location)	96	199
Respiratory rate	95	198
Blood gas	97	193
Heart rate	93	193
General clinical impression	94	186
FiO_2_	88	187
Cyanosis	87	179
Mental status	82	181
Other	10	13

## Supplementary Materials

The following are available online at www.mdpi.com/2227-9032/5/3/34/s1.

## References

[1] Duke T., Blaschke A.J., Sialis S., Bonkowsky J.L. (2002). Hypoxaemia in acute respiratory and non-respiratory illnesses in neonates and children in a developing country. Arch. Dis. Child..

[2] Subhi R., Adamson M., Campbell H., Weber M., Smith K., Duke T., Ashraf H., Berkley J., Bose A., Brent A. (2009). The prevalence of hypoxaemia among ill children in developing countries: A systematic review. Lancet Infect. Dis..

[3] Junge S., Palmer A., Greenwood B.M., Kim Mulholland E., Weber M.W. (2006). The spectrum of hypoxaemia in children admitted to hospital in The Gambia, West Africa. Trop. Med. Int. Health.

[4] Jain D.L., Sarathi V., Jawalekar S. (2013). Predictors of treatment failure in hospitalized children [3–59 months] with severe and very severe pneumonia. Indian Pediatr..

[5] Salah E.T., Algasim S.H., Mhamoud A.S., Husian N.E. (2015). Prevalence of hypoxemia in under-five children with pneumonia in an emergency pediatrics hospital in Sudan. Indian J. Crit. Care Med..

[6] Basnet S., Adhikari R.K., Gurung C.K. (2006). Hypoxemia in children with pneumonia and its clinical predictors. Indian J. Pediatr..

[7] Lozano J.M. (2001). Epidemiology of hypoxaemia in children with acute lower respiratory infection. Int. J. Tuberc. Lung Dis..

[8] Foran M., Ahn R., Novik J., Tyer-Viola L., Chilufya K., Katamba K., Burke T. (2010). Prevalence of undiagnosed hypoxemia in adults and children in an under-resourced district hospital in Zambia. Int. J. Emerg. Med..

[9] Duke T., Mgone J., Frank D. (2001). Hypoxaemia in children with severe pneumonia in Papua New Guinea. Int. J. Tuberc. Lung Dis..

[10] Duke T., Wandi F., Jonathan M., Matai S., Kaupa M., Saavu M., Subhi R., Peel D. (2008). Improved oxygen systems for childhood pneumonia: A multihospital effectiveness study in Papua New Guinea. Lancet.

[11] World Health Organization (WHO) (2013). Pocket Book of Hospital Care for Children: Guidelines for the Management of Common Childhood Illnesses.

[12] Mackway-Jones K., Molyneux E., Phillips B., Wieteska S. (2005). Advanced Paediatric Life Support. The Practical Approach.

[13] Fuchs S., Gauche-Hill M., Yamamoto L. (2007). Advanced Pediatric Life Support. The Pediatric Emergency Medicine Resource.

[14] Forouzanfar M.H., Alexander L., Anderson H.R., Bachman V.F., Biryukov S., Biryukov S., Brauer M., Burnett R., Coates D., GBD 2013 Risk Factors Collaborators (2015). Global, regional, and national comparative risk assessment of 79 behavioural, environmental and occupational, and metabolic risks or clusters of risks in 188 countries, 1990–2013: A systematic analysis for the Global Burden of Disease Study 2013. Lancet.

[15] Rudan I., El Arifeen S., Bhutta Z.A., Black R.E., Brooks A., Chan K.Y., Chopra M., Duke T., Marsh D., Pio A. (2011). Setting research priorities to reduce global mortality from childhood pneumonia by 2015. PLoS Med..

[16] Shefelbine S., Clarkson J., Farmer R., Eason S. (2002). Good Design Practice for Medical Devices and Equipment–Requirements Capture.

[17] Yang K., Ei-Haik B.S. (2008). Design for Six Sigma: A Roadmap for Product Development.

[18] FDA USFaDA, Center for Devices and Radiological Health, Office of Device Evaluation Applying Human Factors and Usability Engineering to Optimize Medical Device Design. https://www.fda.gov/downloads/MedicalDevices/DeviceRegulationandGuidance/GuidanceDocuments/UCM259760.pdf.

[19] Du B., Xi X., Chen D., Peng J. (2010). China Critical Care Clinical Trial G. Clinical review: Critical care medicine in mainland China. Crit. Care.

[20] Wunsch H., Rowan K.M., Angus D.C. (2007). International comparisons in critical care: A necessity and challenge. Curr. Opin. Crit. Care.

[21] Tripathi S., Kaur H., Kashyap R., Dong Y., Gajic O., Murthy S. (2015). A survey on the resources and practices in pediatric critical care of resource-rich and resource-limited countries. J. Intensiv. Care.

[22] The World Bank The World Bank-Data-Country and Lending Groups. http://data.worldbank.org/about/country-and-lending-groups.

[23] Najaf-Zadeh A., Leclerc F. (2011). Noninvasive positive pressure ventilation for acute respiratory failure in children: A concise review. Ann. Intensiv. Care.

[24] Duke T. (2014). CPAP: A guide for clinicians in developing countries. Paediatr. Int. Child Health.

[25] Shelly M.P., Lloyd G.M., Park G.R. (1988). A review of the mechanisms and methods of humidification of inspired gases. Intensiv. Care Med..

[26] Esquinas Rodriguez A.M., Scala R., Soroksky A., BaHammam A., de Klerk A., Valipour A., Chiumello D., Martin C., Holland A.E. (2012). Clinical review: Humidifiers during non-invasive ventilation—Key topics and practical implications. Crit. Care.

[27] Extracorporeal Life Support Organization Center Directory. https://www.elso.org/Members/CenterDirectory.aspx.

[28] Lee K.S., Dunn M.S., Fenwick M., Shennan A.T. (1998). A comparison of underwater bubble continuous positive airway pressure with ventilator-derived continuous positive airway pressure in premature neonates ready for extubation. Biol. Neonate.

[29] Pillow J.J., Hillman N., Moss T.J., Polglase G., Bold G., Beaumont C., Ikegami M., Jobe A.H. (2007). Bubble continuous positive airway pressure enhances lung volume and gas exchange in preterm lambs. Am. J. Respir. Crit. Care Med..

[30] Bahman-Bijari B., Malekiyan A., Niknafs P., Baneshi M.R. (2011). Bubble-CPAP vs. Ventilatory-CPAP in Preterm Infants with Respiratory Distress. Iran. J. Pediatr..

[31] Kawaza K., Machen H.E., Brown J., Mwanza Z., Iniguez S., Gest A., Smith E.O., Oden M., Richards-Kortum R.R., Molyneux E. (2014). Efficacy of a low-cost bubble CPAP system in treatment of respiratory distress in a neonatal ward in Malawi. PLoS ONE.

[32] Chen A., Deshmukh A.A., Richards-Kortum R., Molyneux E., Kawaza K., Cantor S.B. (2014). Cost-effectiveness analysis of a low-cost bubble CPAP device in providing ventilatory support for neonates in Malawi-a preliminary report. BMC Pediatr..

[33] Machen H.E., Mwanza Z.V., Brown J.K., Kawaza K.M., Newberry L., Richards-Kortum R.R., Oden Z.M., Molyneux E.M. (2015). Outcomes of patients with respiratory distress treated with bubble CPAP on a pediatric ward in Malawi. J. Trop. Pediatr..

[34] Jayashree M., KiranBabu H.B., Singhi S., Nallasamy K. (2015). Use of Nasal Bubble CPAP in Children with Hypoxemic Clinical Pneumonia-Report from a Resource Limited Set-Up. J. Trop. Pediatr..

[35] Chisti M.J., Salam M.A., Smith J.H., Ahmed T., Pietroni M.A., Shahunja K.M., Shahid A.S., Faruque A.S., Ashraf H., Bardhan P.K. (2015). Bubble continuous positive airway pressure for children with severe pneumonia and hypoxaemia in Bangladesh: An open, randomised controlled trial. Lancet.

[36] Hill S.E., Njie O., Sanneh M., Jallow M., Peel D., Njie M., Weber M., Hill P.C., Adegbola R.A., Howie S.R. (2009). Oxygen for treatment of severe pneumonia in The Gambia, West Africa: A situational analysis. Int. J. Tuberc. Lung Dis..

[37] Duke T., Graham S.M., Cherian M.N., Ginsburg A.S., English M., Howie S., Peel D., Enarson P.M., Wilson I.H., Were W. (2010). Oxygen is an essential medicine: A call for international action. Int. J. Tuberc. Lung Dis..

[38] Belle J., Cohen H., Shindo N., Lim M., Velazquez-Berumen A., Ndihokubwayo J.B., Cherian M. (2010). Influenza preparedness in low-resource settings: A look at oxygen delivery in 12 African countries. J. Infect. Dev. Ctries..

[39] Ginsburg A.S., Van Cleve W.C., Thompson M.I., English M. (2012). Oxygen and pulse oximetry in childhood pneumonia: A survey of healthcare providers in resource-limited settings. J. Trop. Pediatr..

[40] Floyd J., Wu L., Hay Burgess D., Izadnegahdar R., Mukanga D., Ghani A.C. (2015). Evaluating the impact of pulse oximetry on childhood pneumonia mortality in resource-poor settings. Nature.

[41] World Health Organization (WHO) (2016). Updated Guideline: Paediatric Emergency Triage, Assessment and Treatment.

[42] Weber M.W., Palmer A., Jaffar S., Mulholland E.K. (1996). Humidification of oxygen with unheated humidifiers in tropical climates. Pediatr. Pulmonol..

[43] Nahimana E., Ngendahayo M., Magge H., Odhiambo J., Amoroso C.L., Muhirwa E., Uwilingiyemungu J.N., Nkikabahizi F., Habimana R., Hedt-Gauthier B.L. (2015). Bubble CPAP to support preterm infants in rural Rwanda: A retrospective cohort study. BMC Pediatr..

[44] Hundalani S.G., Richards-Kortum R., Oden M., Kawaza K., Gest A., Molyneux E. (2015). Development and validation of a simple algorithm for initiation of CPAP in neonates with respiratory distress in Malawi. Arch. Dis. Child. Fetal Neonatal. Ed..

[45] Gomersall C.D. (2010). Critical care in the developing world-a challenge for us all. Crit. Care.

[46] Murthy S., Sayeed S.A., Adhikari N.K.J. (2014). Critical Care in Low-Resource Settings.

