# Supplementary material for: Pediatric Respiratory Support Technology and Practices: A Global Survey
Source: Healthcare (Basel). 2017 Jul 21;5(3):34. doi: 10.3390/healthcare5030034 (PMC5618162; doi:10.3390/healthcare5030034)
Supplement: Supplementary file 1 [file healthcare-05-00034-s001.docx]

**Data Supplement: Respiratory Support Technology Survey**

**Pediatric Respiratory Support Technology and Practices : A Global Survey**

**Amélie O. von Saint André-von Arnim, Shelina M. Jamal, Grace C. John-Stewart, Ndidiamaka L. Musa, Joan Roberts, Larissa Stanberry, Christopher Howard**

**Introduction:**

Hello!

Thank you for joining our global landscape assessment of the current non-invasive respiratory support technologies. We appreciate sincerely the time you are taking to consider taking our survey. The survey should take about 10 minutes to complete, depending on your answers. We hope you will answer our questions and choose to stay in touch.

*Who are we?*

We are an international academic research team comprised of researchers, clinicians, designers, and engineers coordinated from Seattle Children’s Research Institute. If you have comments or questions about our work, you can contact us using this email address: respsupportsurvey@seattlechildrens.org. We will do our best to respond to you in 48 hours.

*How will we use the results from this survey?*

We expect to publish the results and provide the de-identified raw data to the global community for further analysis.

We expect also to use the data to guide design and development priorities for durable non-invasive pediatric respiratory support systems that are suitable for less-resourced healthcare settings.

How do you navigate the survey?

Please use the navigation buttons at the bottom of each page for going forwards and backwards in the survey. Don’t use the back button in the browser to return to pages you have already completed.

*IRB review*

This survey has been reviewed by the Seattle Children's Institutional Review Board with an exempt determination. Your responses are anonymous, unless you choose to leave your name and email address at the end.

Let's go...

**Section 1: Questions about your background**

*What is your primary job title / role?*

- Nurse - General
- Nurse - Critical care
- Nurse - Pediatric
- Nurse - Pediatric critical care
- Nurse - Pediatric emergency medicine
- Nurse - Trainee
- Physician - Anesthesiologist
- Physician - Critical care physician
- Physician - Emergency medicine physician
- Physician - General practitioner
- Physician - Pediatric critical care physician
- Physician - Pediatric emergency medicine physician
- Physician - Pediatrician
- Physician - Surgeon
- Physician - Trainee (Medical student, resident, fellow)
- Respiratory Therapist
- Other - Please specify:

You listed "Other" above. Please provide you job title and role.

*Which country do you work in?*

| Afghanistan | China | Guyana | Mali | San Marino | Ukraine |
| --- | --- | --- | --- | --- | --- |
| Albania | Colombia | Haiti | Malta | São Tomé and Principe | United Arab Emirates |
| Algeria | Comoros | Honduras | Marshall Islands | Saudi Arabia | United Kingdom |
| American Samoa | Congo - Dem. Rep | Hong Kong SAR - China | Mauritania | Senegal | United States |
| Andorra | Congo - Rep. | Hungary | Mauritius | Serbia | Uruguay |
| Angola | Costa Rica | Iceland | Mexico | Seychelles | Uzbekistan |
| Antigua and Barbuda | Côte d'Ivoire | India | Micronesia - Fed. Sts. | Sierra Leone | Vanuatu |
| Argentina | Croatia | Indonesia | Moldova | Singapore | Venezuela - RB |
| Armenia | Cuba | Iran - Islamic Rep. | Monaco | Sint Maarten | Vietnam |
| Aruba | Curaçao | Iraq | Mongolia | Slovak Republic | Virgin Islands (U.S.) |
| Australia | Cyprus | Ireland | Montenegro | Slovenia | West Bank and Gaza |
| Austria | Czech Republic | Isle of Man | Morocco | Solomon Islands | Yemen - Rep. |
| Azerbaijan | Denmark | Israel | Mozambique | Somalia | Zambia |
| Bahamas - The | Djibouti | Italy | Myanmar | South Africa | Zimbabwe |
| Bahrain | Dominica | Jamaica | Namibia | South Sudan | Not Listed |
| Bangladesh | Dominican Republic | Japan | Nepal | Spain |  |
| Barbados | Ecuador | Jordan | Netherlands | Sri Lanka |  |
| Belarus | Egypt - Arab Rep. | Kazakhstan | New Caledonia | St. Kitts and Nevis |  |
| Belgium | El Salvador | Kenya | New Zealand | St. Lucia |  |
| Belize | Equatorial Guinea | Kiribati | Nicaragua | St. Martin |  |
| Benin | Eritrea | Korea - Dem Rep. | Niger | St. Vincent and the Grenadines |  |
| Bermuda | Estonia | Korea - Rep. | Nigeria | Sudan |  |
| Bhutan | Ethiopia | Kosovo | Northern Mariana Islands | Suriname |  |
| Bolivia | Faeroe Islands | Kuwait | Norway | Swaziland |  |
| Bosnia and Herzegovina | Fiji | Kyrgyz Republic | Oman | Sweden |  |
| Botswana | Finland | Lao PDR | Pakistan | Switzerland |  |
| Brazil | France | Latvia | Palau | Syrian Arab Republic |  |
| Brunei Darussalam | French Polynesia | Lebanon | Panama | Tajikistan |  |
| Bulgaria | Gabon | Lesotho | Papua New Guinea | Tanzania |  |
| Burkina Faso | Gambia - The | Liberia | Paraguay | Thailand |  |
| Burundi | Georgia | Libya | Peru | Timor-Leste |  |
| Cabo Verde | Germany | Liechtenstein | Philippines | Togo |  |
| Cambodia | Ghana | Lithuania | Poland | Tonga |  |
| Cameroon | Greece | Luxembourg | Portugal | Trinidad and Tobago |  |
| Canada | Greenland | Macao SAR - China | Puerto Rico | Tunisia |  |
| Cayman Islands | Grenada | Macedonia - FYR | Qatar | Turkey |  |
| Central African Republic | Guam | Madagascar | Romania | Turkmenistan |  |
| Chad | Guatemala | Malawi | Russian Federation | Turks and Caicos Islands |  |
| Channel Islands | Guinea | Malaysia | Rwanda | Tuvalu |  |
| Chile | Guinea-Bisau | Maldives | Samoa | Uganda |  |

(If you work in more than one country, indicate which country you work in primarily.)

*What setting is your work facility based in?*

- Urban
- Suburban
- Rural
- Other - Please specify:

(If you work in more than one facility, indicate the setting of the facility you work in primarily.)

If you listed "Other" above, please describe the setting of healthcare facility you work in primarily.

*What type of healthcare facility do you work in primarily? Click all that apply.*

- Public
- Private
- Faith-based
- Other - Please specify:

If you listed "Other" above. Please briefly describe type of healthcare facility you work in primarily.

*What level of healthcare facility do you work in primarily?*

- Primary level care, clinic or community hospital
- Secondary level care or district hospital
- Tertiary level care or regional referral center or University hospital
- Other - Please specify

You listed "Other" above. Please briefly describe level of healthcare facility you work in primarily.

*How many beds does your facility have approximately?*

- Typed in integer number

(If you do not know, please enter "Do not know", "Don't know", "DNK", etc.)

*What ages does your facility treat? Check all that apply.*

- Premature Infants and Neonates (up to 28 days)
- 28 days to 12 years.
- 12 years to 18 years
- 18 years and above

*What types of beds does your facility have? Check all that apply*.

- Neonatal ward (non-ICU) beds
- Neonatal ICU beds
- Pediatric ward (non-ICU) beds Pediatric (non-neonatal) ICU beds
- Mixed pediatric and adult ward (non-ICU) beds Mixed pediatric and adult ICU beds
- Adult ward (non-ICU) beds Adult ICU beds

*How many neonatal ward (non-ICU) beds does your facility have?*

- Typed in integer number

(If you do not know, please enter "Do not know", "Don't know", "DNK", etc.)

*How many neonatal ICU beds does your facility have?*

- Typed in integer number

(If you do not know, please enter "Do not know", "Don't know", "DNK", etc.)

*How many pediatric ward (non-ICU) beds does your facility have?*

- Typed in integer number

(If you do not know, please enter "Do not know", "Don't know", "DNK", etc.)

*How many pediatric ICU beds does your facility have?*

- Typed in integer number

(If you do not know, please enter "Do not know", "Don't know", "DNK", etc.)

*How many mixed pediatric and adult ICU beds does your facility have?*

- Typed in integer number

(If you do not know, please enter "Do not know", "Don't know", "DNK", etc.)

*How many mixed pediatric and adult ward (non-ICU) beds does your facility have?*

- Typed in integer number

(If you do not know, please enter "Do not know", "Don't know", "DNK", etc.)

*How many adult ward (non-ICU) beds does your facility have?*

- Typed in integer number

(If you do not know, please enter "Do not know", "Don't know", "DNK", etc.)

*How many adult ICU) beds does your facility have?*

- Typed in integer number

(If you do not know, please enter "Do not know", "Don't know", "DNK", etc.)

**Section 2: Questions about your current respiratory support capabilities**

*How do you manage children with acute respiratory failure? Check all that apply.*

- Maximize supplemental oxygen
- Bag-mask ventilation
- High flow nasal cannula support
- Non-invasive positive pressure support
- Intubation and manual ventilation Intubation and mechanical ventilation
- Transport to a higher level of care
- ECMO
- Other - Please specify:

You listed "Other" above. Please briefly describe how you manage children with acute respiratory failure.

*What non-invasive respiratory support do you use for children? Check all that apply*.

- Bilevel Positive Airway Pressure (BiPAP)
- Bubble CPAP
- Continuous Positive Airway Pressure (CPAP)
- High flow nasal cannula (HFNC)
- Oxygen via facemask or nasal cannula
- Non-invasive positive pressure respiratory support or HFNC are not available at our facility
- Other - Please specify:

You indicated you are using high flow nasal cannula (HFNC). What is the maximum flow rate (liters per min) you are able to deliver?

You listed "Other" above. Please briefly describe what non-invasive respiratory support you use for children.

**Section 3: Questions about the facility you work in primarily**

*How many patients do you care for in a normal work day or shift?*

- Typed in integer number

(If you do not know, please enter "Do not know", "Don't know", "DNK", etc)

*Where does care of acutely-ill pediatric patients Emergency room or urgent care take place in your facility? Check all that apply.*

- Neonatal Intensive care unit (NICU) Pediatric Intensive care unit (PICU)
- High dependency/ close observation or step-down unit
- Pediatric ward
- Other location - Please specify

You listed "Other" above. Please name or briefly describe the location where care of acutely-ill patients takes place.

*How many patients does one nurse care for normally in* the Emergency Room at your facility? (e.g. 10)

- Typed in integer number

(If you do not know, please enter "Do not know", "Don't know", "DNK", etc.)

*How many patients does one doctor (physician) care for normally in the Emergency Room at your facility? (e.g. 30)*

- Typed in integer number

(If you do not know, please enter "Do not know", Don't know", "DNK", etc.)

*How many patients does one nurse care for normally in the Neonatal Intensive Care Unit (NICU) at your facility? (e.g. 10)*

(If you do not know, please enter "Do not know", "Don't know", "DNK", etc.)

*How many patients does one doctor (physician) care for normally in the Neonatal Intensive Care Unit at your facility? (e.g. 30)*

- Typed in integer number

(If you do not know, please enter "Do not know", (NICU) "Don't know", "DNK", etc.)

*How many patients does one nurse care for normally in the Pediatric Intensive Care Unit (PICU) at your facility? (e.g. 10*)

- Typed in integer number

(If you do not know, please enter "Do not know", "Don't know", "DNK", etc.)

*How many patients does one doctor (physician) care for in the Pediatric Intensive Care Unit (PICU) at your facility (e.g. 30)?*

- Typed in integer number

(If you do not know, please enter "Do not know", "Don't know", "DNK", etc.)

*How many patients does one nurse care for normally in the High Dependency / Close Observation Unit at your facility? (e.g. 10)*

- Typed in integer number

(If you do not know, please enter "Do not know", "Don't know", "DNK", etc.)

*How many patients does one doctor (physician) care for normally in the High Dependency / Close Observation Unit at your facility?*

- Typed in integer number

(If you do not know, please enter "Do not know", (e.g. 30) "Don't know", "DNK", etc.)

*How many patients does one nurse care for normally in the Pediatric Ward at your facility? (e.g. 10)*

- Typed in integer number

(If you do not know, please enter "Do not know", "Don't know", "DNK", etc.)

*How many patients does one doctor (physician) care for normally in the Pediatric Ward at your facility? (e.g. 30)*

- Typed in integer number

(If you do not know, please enter "Do not know", "Don't know", "DNK", etc.)

*How many patients does one nurse care for normally in the other location at your facility you refer to above? (e.g. 10)*

- Typed in integer number

(If you do not know, please enter "Do not know", "Don't know", "DNK", etc.)

How many patients does one doctor (physician) care for normally in the other location at your facility you refer to above? (e.g. 30)

- Typed in integer number

(If you do not know, please enter "Do not know", "Don't know", "DNK", etc.)

*Do you have the following connections at your facility?*

| Cellular / mobile phone | Yes and it is reliable | Yes but it is unreliable | No | Don't know / Not sure |
| --- | --- | --- | --- | --- |
| Cellular / mobile data Wired Internet | Yes and it is reliable | Yes but it is unreliable | No | Don't know / Not sure |
| Wireless Internet (Wifi) | Yes and it is reliable | Yes but it is unreliable | No | Don't know / Not sure |

*How many hours a day does your facility have electricity approximately?*

- Drop down box 0 – 24
- Don't know / Not Sure

*How do you record clinical parameters/ vital signs? Check all that apply.*

- Paper records
- Electronic records
- We don't record clinical parameters
- Other. Please specify
- Don't know / Not sure

You checked "Other" above. Please describe how you record clinical parameters/vital signs?

*Are the following respiratory system components for non-invasive respiratory support available and in regular use at your facility? Please check all that apply.*

| Oxygen source Air source | Yes | No | Don't know / Not sure |
| --- | --- | --- | --- |
| Gas blender | Yes | No | Don't know / Not sure |
| Gas humidifier / heater Breathing circuit / tubing | Yes | No | Don't know / Not sure |
| Patient interface (e.g. face mask, nasal prongs) | Yes | No | Don't know / Not sure |
| Positive pressure ventilation system (e.g. CPAP, BiPAP, HFNC, ventilator) | Yes | No | Don't know / Not sure |
| Oxygen saturation monitor (e.g. pulse oximeter) | Yes | No | Don't know / Not sure |

*What is the source(s) of oxygen at your facility? Check all that apply.*

- Wall outlet
- Bottles
- Oxygen concentrator
- Other
- Don't know / Not sure

*What the source(s) of air at your facility? Check all that apply.*

- Wall outlet
- Bottles
- Compressor Electric pump
  Other
- Don't know / Not sure

*What type of Humidifier do you use at your facility? Check all that apply.*

| Heated humidifier | Bubble / bottle humidifier | Pass over / wick humidifier | HME (Heat and moisture exchanger) | Other - Please specify: | Don't know / Not sure |
| --- | --- | --- | --- | --- | --- |
| Unheated humidifier | Bubble / bottle humidifier | Pass over / wick humidifier | HME (Heat and moisture exchanger) | Other - Please specify: | Don't know / Not sure |

You checked "Other" above. Please describe the type of humidifier you use.

*What type of patient interface do you use at your facility? Check all that apply.*

- Nasopharyngeal cannula (one nostril)
- Nasal prongs (two nostril)
- Nasal mask (over the nose)
- Face mask (over the face)
- Other - Please specify: Don't know / Not sure

You checked "Other" above. Please describe the type of patient interface you use.

What type of positive pressure ventilation do you use at your facility? Check all that apply.

- Ventilator
- Dedicated CPAP/BiPAP machine
- Bubble CPAP
- High Flow Nasal Cannula
- Other - Please specify:
- Don't know / Not sure

You checked "Other" above. Please describe the type of positive pressure ventilation you use.

Please rate the importance of the following factors that promote the ongoing use of new equipment/technology at your facility? (0 = Not important, 4 = Very important)

|  | 0 (Not important) | 1 | 2 (Somewhat import) | 3 | 4 (Very important) |
| --- | --- | --- | --- | --- | --- |
| Scientific (published) clinical evidence |  |  |  |  |  |
| Personal experience |  |  |  |  |  |
| Adequate training and support of doctors and nurses |  |  |  |  |  |
| Workload of medical staff |  |  |  |  |  |
| Use is required by a supervisor or manager |  |  |  |  |  |
| User-friendliness of the equipment |  |  |  |  |  |
| Initial equipment cost |  |  |  |  |  |
| Ongoing costs (electricity, consumables, maintenance costs) |  |  |  |  |  |
| Ongoing technical/maintenance support |  |  |  |  |  |
| Reusability of all equipment components |  |  |  |  |  |
| Durability of the equipment |  |  |  |  |  |
| Equipment safety |  |  |  |  |  |
| Other - Please specify: |  |  |  |  |  |

You listed "Other" above. Please describe any other factors that may limit the adoption of new equipment/technology at your facility.

*Do you track hospital acquired infections?*

- Yes
- No
- Don't know / Not sure

*Have you successfully implemented standardized clinical protocols for management of common acute illness?*

- Yes
- No
- Don't know / Not sure

*If yes, which methods were most helpful for implementing standardized clinical protocols at your facility?*

- Adequate training and introduction to the new practice
- Ongoing support for practice change
- Availability of necessary equipment
- Decreased workload of doctors and / or nurses Required by the hospital/department
- Other - Please specify:

You listed "Other" above. Please describe which resources were most helpful for protocol implementation?

**Section 4: Questions about respiratory support measurements and evaluation.**

*How do you assess severity in children with respiratory diseases? Check all that apply.*

- General clinical impression
- Work of breathing (grunting, flaring, tracheal tugging)
- Chest retractions (degree, location) Respiratory rate
- Oxygen saturation Cyanosis
- Heart rate Mental status
- Fraction of inspired oxygen (FiO2) Blood gas
- Other - Please specify:

You listed "Other" above. Please list the other way or ways you assess severity.

*Which clinical parameters are you charting? Check all that apply.*

- Respiratory rate (RR)
- Fraction of inspired oxygen (FiO2)
- Oxygen saturation Chest retractions Dyspnea
- Cyanosis Heart rate
- Auscultatory findings
- Mental status
- Respiratory score
- Other, please specify

You listed "Other" above. Please list the other clinical parameters you are charting.

*At your highest level of care (e.g. ICU), how often do you chart these parameters?*

|  | Hourly | Every 2 hours | Every 4 hours | Every 6 hours | Every 8 hours | Every 12 hours | Daily (Every 24 hours) | Other Don't know / Not sure |
| --- | --- | --- | --- | --- | --- | --- | --- | --- |
| Respiratory rate |  |  |  |  |  |  |  |  |
| Fraction of inspired oxygen (FiO2) |  |  |  |  |  |  |  |  |
| Oxygen saturation |  |  |  |  |  |  |  |  |
| Chest retractions Dyspnea Cyanosis |  |  |  |  |  |  |  |  |
| Heart rate |  |  |  |  |  |  |  |  |
| Auscultatory findings |  |  |  |  |  |  |  |  |
| Mental status |  |  |  |  |  |  |  |  |
| Respiratory score |  |  |  |  |  |  |  |  |
| Fraction of inspired oxygen (FiO2) |  |  |  |  |  |  |  |  |

You listed "Other" on the previous page. Please list other parameters you chart and how often you chart the each parameter

**Section 5 (Optional): Respiratory Score Details**

*You mentioned you used a respiratory score earlier.*

Which respiratory score are you using?

- Typed in name

(If you are using an internally developed score please enter "internal". If you do not know, please enter "Do not know", "Don't know", "DNK", etc.)

*Which parameters does your respiratory score include? Check all that apply.*

- Respiratory rate.
- Degree of chest retractions.
- Oxygen saturation.
- Cyanosis.
- Heart rate.
- Auscultation findings
- Mental status.
- Work of breathing.
- General clinical impression.
- Fraction if inspired oxygen (FiO2)
- Other - please specify:
- Don't know / Not sure

You checked "Other" above. Please provide the other parameters your respiratory score includes

**End Section:**

The end!

Thank you for taking the time to complete our survey.

If you have any anonymous comments or concerns about this survey, please leave them in the comments box at the bottom of this page.

If you would like to stay in touch, receive updates on this survey, or have questions or comments that need a direct response from us, the following new page enables you to leave your name and/or email address. Your contact information is not required and is not associated with your survey responses. We will not share your contact information with 3rd parties, unless required to do so by law.

If you would like to reach out to us directly, you can also send an email to the following address and we will try to respond to you in 48 hours. Our email address is: respsupportsurvey@seattlechildrens.org

Sincerely,

The Pediatric Respiratory Support Research Team

**Separate Identifiable Comments and Contact Info Page:**

If you would like to stay in touch, receive updates on this survey, or have questions or comments that need a direct response from us, please leave your name and/or email address.

- Your name:
- Your email address:
- Please add comments or questions that you would like a direct answer or response to:

Your contact information is not required and is not associated with your survey responses. We will not share your contact information with 3rd parties, unless required to do so by law.

Thank you for your time. Sincerely,

The Pediatric Respiratory Support Research Team
